# Effectiveness of walking versus mind-body therapies in chronic low back pain: A systematic review and meta-analysis of recent randomized controlled trials

**DOI:** 10.1097/MD.0000000000021969

**Published:** 2020-08-28

**Authors:** Ildephonse Nduwimana, Félix Nindorera, Jean Louis Thonnard, Oyene Kossi

**Affiliations:** a Institute of Neuroscience, Université Catholique de Louvain, Brussels, Belgium; b National Center for Physical Therapy and Rehabilitation (CNRKR), Bujumbura, Burundi; c Department of Rehabilitation, University Hospital of Parakou; d National School of Public Health and Epidemiology, University of Parakou, Parakou, Bénin.

**Keywords:** chronic low back pain, meta-analysis, mind-body therapy, systematic review, walking

## Abstract

**Purpose:**

Walking and mind-body therapies (MBTs) are commonly recommended to relieve pain and improve function in patients with chronic low back pain (CLBP). The purpose of this study was to compare the effectiveness of walking and MBTs in CLBP.

**Methods:**

We included randomized controlled trials (RCTs) comparing walking or MBTs to any other intervention or control in adults with CLBP. Studies were identified through PubMed, Cochrane Library, PsycINFO, Scopus, and ScienceDirect databases. The research was limited to studies published in English and French between January 2008 and December 2018. Two reviewers independently selected the studies, extracted data, and assessed studies quality using the Physiotherapy Evidence Database (PEDro) scale. Statistical analyses were performed under a random-effects model. We analyzed pain and activity limitation, with the calculation of standardized mean differences and 95% confidence intervals for the different treatment effects.

**Results:**

Thirty one randomized controlled trials involving 3193 participants were analyzed. Walking was as effective as control interventions in the short-term and slightly superior in the intermediate term with respect to pain (Standardized mean differences (SMD)  = –0.34; 95% CI, –0.65 to –0.03; *P* = .03) and activity limitation (SMD = –0.30; 95% CI, –0.50 to –0.10; *P* = .003). In contrast, yoga was more effective than control interventions in the short term in terms of pain (SMD = –1.47; 95% CI, –2.26 to –0.68; *P* = .0003) and activity limitation (SMD = –1.17; 95% CI, –1.80 to –0.55; *P* = .0002). Yoga was no longer superior to the control interventions for pain at the 6-month follow-up.

**Conclusion:**

MBTs, especially yoga, seem to be more effective in the short term, and walking seems to be more effective in the intermediate term, for the relief of pain and activity limitation in patients with CLBP. A combination of walking and MBTs fits the biopsychosocial model and might be valuable therapy for CLBP throughout follow-up due to combined effects.

## Abstract 2

Supplemental Digital Content is available in the text

## 1 Introduction

Low back pain (LBP) is a common symptom worldwide in all age groups, mainly in working populations.^[1,2]^ Globally, LBP results in more years lived with disability than does any other health condition; its global burden is projected to increase, especially in low-income and middle-income countries, due to population increases, informal employment, and fragile health systems.^[1,3]^ Non-specific low back pain (NSLBP) is defined as LBP that is not attributable to a known specific pathology. It can be acute, subacute, or chronic. Chronic low back pain (CLBP) has a duration of >3 months, or occurs episodically within a 6-month period.^[4]^ Meucci et al^[5]^ reported that the prevalence of CLBP was 19.6% among individuals aged 20 to 59 years (an actively working age group). Management guidelines for this condition recommend graded activity or exercise programs and psychosocial interventions.^[6]^ Among such interventions, mind-body therapies (MBTs) are cited as an option to improve function and reduce pain.^[7]^ The United States National Center for Complementary and Alternative Medicine defined MBTs as healing techniques that enhance the minds capacity to affect bodily functions and symptoms.^[8]^ These therapies induce relaxation and improve overall health and well-being. Yoga is the most widely used MBT for CLBP management and has been demonstrated to effectively improve back-related function.^[9]^ Apart from MBTs, walking is cited as an active exercise that reduces pain and activity limitations in patients with CLBP.^[10]^ To our knowledge, no systematic review or meta-analysis has been performed to compare the effectiveness of walking and MBTs for CLBP. Therefore, the aim of this study was to review the literature and to compare the effectiveness of these 2 therapies in the management of CLBP.

## 2 Methods

### 2.1 Study protocol

This systematic review and meta-analysis were performed according to our previous protocol, which was registered in the international prospective register of systematic reviews, PROSPERO (https://www.crd.york.ac.uk/PROSPERO; no. CRD42018092239). The study complied with the Preferred Reporting Items for Systematic Reviews and Meta-Analyses statement.^[11]^ Methodological issues were resolved with guidance from the Cochrane Handbook for Systematic Reviews of Interventions.^[12]^ This systematic review and meta-analysis did not require formal ethical approval because all data were analyzed anonymously

### 2.2 Data sources and searches

We searched 5 electronic databases (MEDLINE/PubMed, Cochrane Library, Scopus, PsycINFO, and ScienceDirect) for articles published in English or French from January 2008 to December 2018. The search strategy combined terms related to or describing walking and/or MBTs for LBP. The search was adapted to each database, with combinations of keywords and Medical Subject Headings (MeSH) terms used as applicable. Additionally, published reviews and the reference lists of retrieved publications were searched manually. The full search strategy used for each database is presented in Appendix 1.

### 2.3 Study selection

#### 2.3.1 Studies and participants

Included studies were randomized controlled trials (RCTs) comparing walking or MBT to any other intervention or control treatment. To be included, studies had to report on adults (age ≥ 18 years) diagnosed with LBP lasting for ≥3 months. Studies involving patients with specific CLBP were excluded. We also excluded studies for which the effect of walking or MBT could not be isolated because the activity was part of a multidisciplinary program.

#### 2.3.2 Interventions and comparators

The trials considered in this study used walking, yoga, tai chi, qigong, and mindfulness meditation as experimental interventions for CLBP. Considering that, with the exception of 1 study, the RCTs did not compare walking with MBTs, our analysis considered walking as the intervention and MBTs as the comparator.

#### 2.3.3 Description of the intervention

Walking is the most popular type of moderate-intensity physical activity. Walking exercise is associated with lower rates of chronic diseases, and it improves patients physical capacity and thus their functional autonomy. It can be performed as over ground walking with or without a pedometer, as step walking, or as treadmill walking.^[13]^

#### 2.3.4 Comparators and controls

MBTs are defined as treatment methods or techniques that are based on mind–body interactions. They can be used to reduce biomechanical and psychological symptoms, and to enhance individuals physiological and psychological well-being.^[8]^ MBTs include tai chi (ji), yoga, qigong, meditation, mental healing, and relaxation therapy.

#### 2.3.5 Outcome measures

In the included studies, LBP was evaluated using visual analog scales, numerical rating scales, and other scoring systems. Activity limitation was evaluated using the Oswestry Disability Index, the Roland and Morris Disability Questionnaire, and other measures. When available, we extracted data obtained at baseline, after the intervention, and at all reported follow-up time points, classified as short-term (0–3 months after the intervention), intermediate-term (3–6 months post-intervention) and long-term (>6 months post-intervention).

### 2.4 Data collection and analysis

#### 2.4.1 Data extraction and quality assessment

The first and second authors independently screened the titles and abstracts of all unique records for relevance. Full texts of selected papers were reviewed and data were extracted using an Excel (Microsoft Corporation, Redmond, WA, USA) spreadsheet. The authors agreement on study selection and rating was assessed, and differences were discussed until consensus was reached. When necessary, a third author was consulted. Two reviewers assessed the risk of bias of included studies using the Physiotherapy Evidence Database (PEDro) scale,^[14]^ an 11-item scale designed for rating of the methodological quality of randomized trials. Each item except item 1 can contribute 1 point to the total PEDro score (1 = satisfied, 0 = not satisfied; maximum = 10 points); item 1 is related to the external validity or generalizability of the sample. In case of disagreement between the reviewers, a third reviewer was consulted.

#### 2.4.2 Assessment of heterogeneity

Heterogeneity refers to clinical, methodological, and statistical differences among studies. We assessed heterogeneity using the *I*^2^ statistic with associated *P* values. The *I*^2^ value describes the percentage of variability in the effect estimate that is due to clinical or methodological heterogeneity, rather than to chance. *I*^2^ values of 30% to 60% represent moderate heterogeneity, values of 50% to 90% represent substantial heterogeneity, and values of 75% to 100% represent considerable heterogeneity.^[15]^ A significant *P* value indicates a lack of homogeneity of findings. Tau-Squared values were used to determine the variance of study effects and associated degrees of freedom.

#### 2.4.3 Data synthesis and analysis

To determine the most effective interventions for the treatment of CLBP, exploratory subgroup analyses were conducted based on intervention type. Statistical analyses were performed using a random-effects model with Review Manager Software (version 5.3).^[15]^ Standardized mean differences (SMDs) with 95% confidence intervals (CIs) were calculated. The SMD reflects the intervention effect size (ES) in each study relative to the variability observed in that study. An SMD of 0 means that the treatment and control (or placebo) have equivalent effects. Improvement is associated with higher scores on the outcome measure, SMDs >0 or <0 indicate the degree to which the treatment is more or less effective, respectively, than the control (or placebo).^[16]^ ESs were calculated based on means and standard deviations, and on the sizes of the intervention and control groups. ESs calculated with SMDs were interpreted using Cohens method and classified as small (0.20), medium (0.50), and large (0.80).^[17]^

#### 2.4.4 Sensitivity analysis

A pre-set cut-off point of ≥50% was used to select trials for the sensitivity analysis. We assessed the effect of the cut-off point used in the methodological quality assessment on the level of evidence with the PEDro scale. We assessed how sensitive the results of the review were in relation to quality of included trials. Sensitivity analysis of the synthesized results was conducted to explore the impact of excluding studies from the meta-analysis based on methodological quality. Only yoga and walking trials of moderate to high quality (PEDro score ≥5) were analyzed.

## 3 Results

### 3.1 Description of studies

#### 3.1.1 Included studies

**Figure 1:**
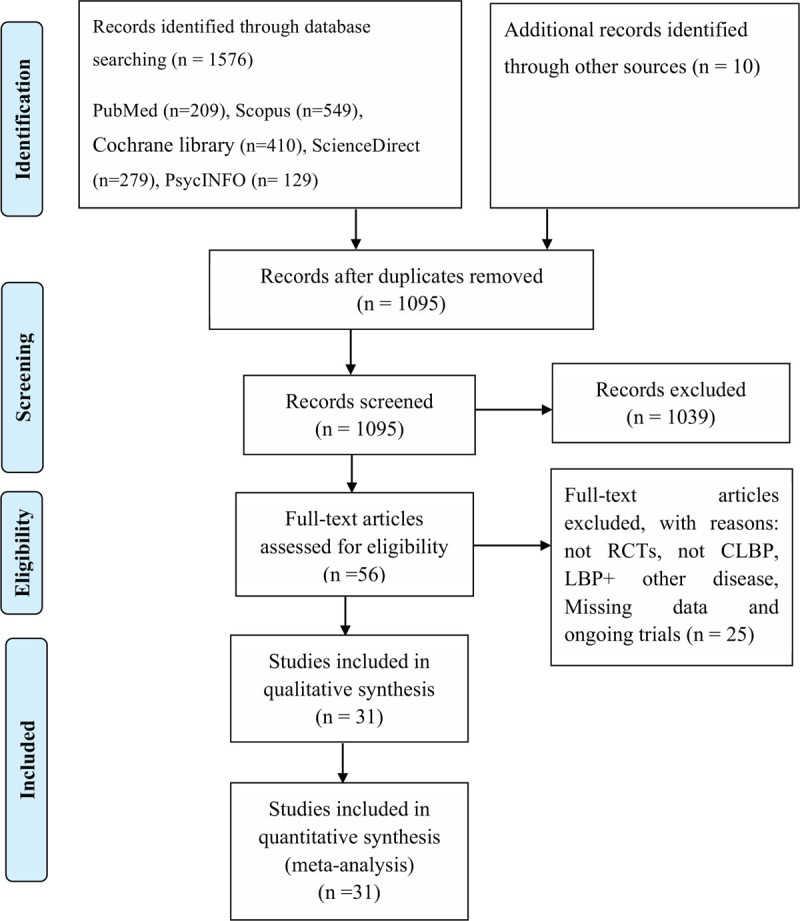
The PRISMA flow diagram of inclusion.

We identified 1576 records of possible interest in the electronic database searches. Ten additional records were identified from other sources (publications references, other systematic reviews, and meta-analyses). After duplicate removal, screening of titles and abstracts, and review of full texts, 31 RCTs were determined to meet the inclusion criteria (Fig. 1). The 31 studies involved 3193 participants, with sample sizes ranging from 20 to 320.

#### 3.1.2 Study characteristics

The characteristics of the 31 studies included in this review are described in Table 1. We found only 1 RCT comparing walking with MBT; 30 RCTs examined the effectiveness of walking and MBTs separately. Twelve studies were conducted in Asia (India, Korea, Israel, Turkey, China, and Thailand), 8 studies each were performed in the USA and Europe (Germany, United Kingdom, Denmark, Ireland, and Croatia), and 1 trial each was conducted in South America (Brazil), Africa (Nigeria), and Australia. One trial^[18]^ assessed yoga and qigong interventions and was thus included in both subgroups. Intervention durations ranged from 7 days to 12 months. Thirty one studies involved short-term follow-up and 10 involved intermediate-term follow-up. Trials were classified into 5 subgroups: walking (*n* = 10), yoga (*n* = 13), tai chi (*n* = 2), meditation (*n* = 4), and qigong (*n* = 3).

**Table 1:**
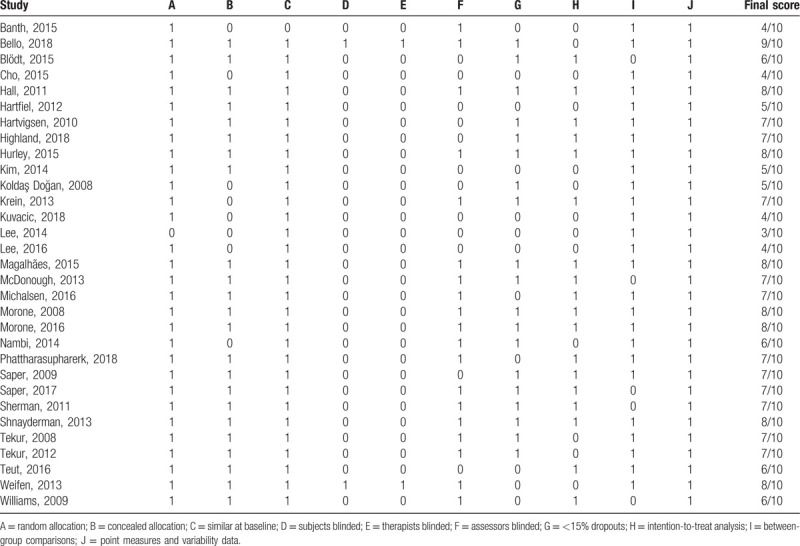
PEDro scores.

### 3.2 Methodological quality

The majority (74.2%) of the included trials^[18–20,22,23,25,26,28,30,31,34–38,40–45,47,48]^ were of moderate to high quality (PEDro score ≥ 6; Table 1). Only 2 trials^[19,38]^ involved the blinding of subjects and therapists to the treatments; 19 (61.2%) RCTs^[19,22,23,25–27,30,31,34–38,40–42,45,47,48]^ involved assessor blinding.

### 3.3 Subgroup analyses

#### 3.3.1 Short-term effects of interventions on pain

**Figure 2:**
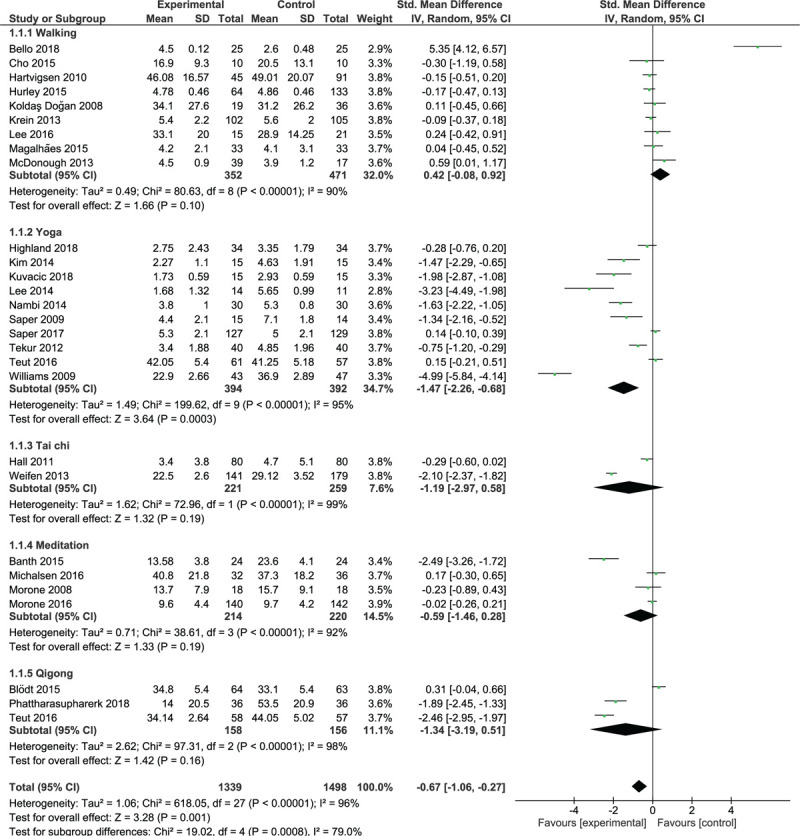
Short-term effect of walking and mind-body therapies on pain.

Nine trials involving 823 participants were included in the walking trials subgroup (Fig. 2). The overall analysis demonstrated a non-significant effect and a high degree of heterogeneity (*I*^2^ = 90%, *P* < .00001); walking and control interventions were equally effective for pain reduction in the short term. Four studies^[29,30,35,43]^ yielded non-significant results in favor of walking, 4 trials^[24,31,36,46]^yielded non-significant results in favor of control interventions, and 1 trial^[20]^ showed that lumbar stabilization exercises were statistically superior to treadmill walking exercise for the reduction of pain.

Ten trials with 786 participants were included in the yoga trials subgroup (Fig. 2). The analysis showed a large significant effect (SMD = –1.47; 95% CI, –2.26 to –0.68; *P* = .0003) and a high degree of heterogeneity (*I*^2^ = 95%, *P* < .00001); yoga interventions were more effective than control interventions in terms of pain improvement in the short term. Eight of the 10 trials yielded results favoring yoga over the control interventions, which were significant in 6 trials.^[21,32–34,40,45]^ Intervention frequency was reported for 4 of these 6 trials as twice per week,^[45]^ 3 times per week,^[32,33]^ and every day.^[40]^

Two trials with 480 participants were included in the tai-chi trials subgroup (Fig. 2). These trials yielded results in favor of tai chi, but significance was achieved in only 1 trial.^[38]^ The overall analysis demonstrated a non-significant effect (*P* = .19) and a high degree of heterogeneity (*I*^2^ = 99%, *P* < .00001).

Four trials with 434 participants were included in the meditation trials subgroup (Fig. 2). Three trials yielded results in favor of meditation, but significance was achieved in only 1 trial.^[27]^ The overall analysis demonstrated no significant effect (*P* = .19) and a high degree of heterogeneity (*I*^2^ = 92%, *P* < .00001); meditation was as effective as control interventions.

Three trials with 314 participants were included in the qigong trials subgroup (Fig. 2). Two trials^[18,22]^ yielded significant results in favor of qigong. The overall analysis demonstrated no significant effect (*P* = .16) and a high degree of heterogeneity (*I*^2^ = 98%, *P* < .00001); qigong was as effective as the control interventions. Analysis of the pooled effect of walking and MBTs showed a significant moderate effect (SMD = –0.67; 95% CI, –1.06 to –0.27; *P* = .001) in favor of these therapies over control treatments.

#### 3.3.2 Short-term effects of interventions on activity limitation

**Figure 3:**
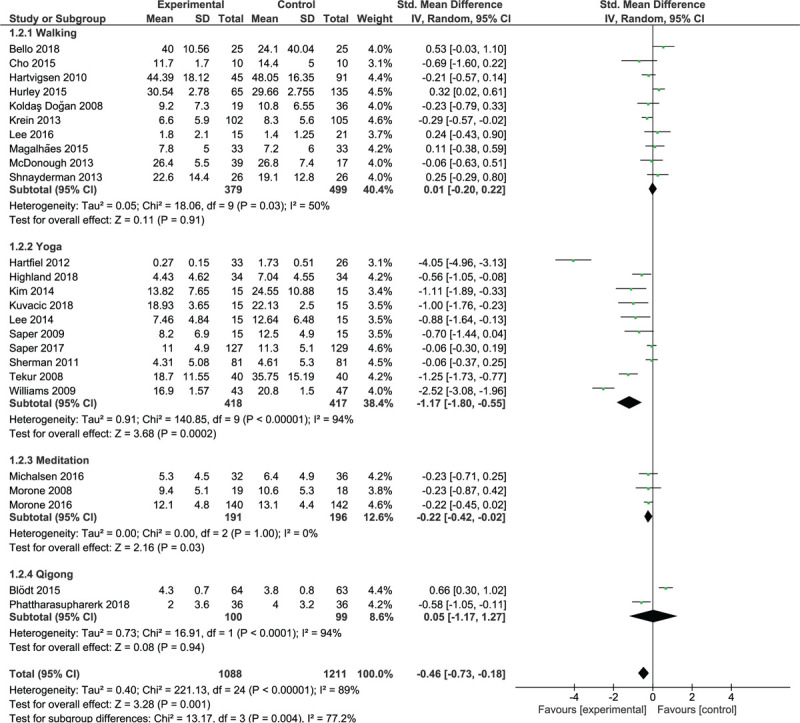
Short-term effect of walking and MBT on activity limitations.

Ten trials involving 878 participants were included in the walking trials subgroup (Fig. 3). The overall analysis demonstrated a non-significant effect (*P* = .91) and a moderate degree of heterogeneity (*I*^2^ = 50%, *P* = .03); walking interventions were as effective as control interventions in reducing activity limitation in the short term. Five trials^[29,35,36,43,46]^ yielded non-significant results in favor of walking, and 5 trials^[19,24,30,31,37]^ yielded non-significant results in favor of control interventions.

Ten trials with 835 participants were included in the yoga trials subgroup (Fig. 3). The analysis demonstrated a large significant effect (SMD = –1.17; 95% CI, –1.80 to –0.55; *P* = .0002) and a high degree of heterogeneity (*I*^2^ = 94%, *P* < .00001). All trials yielded results in favor of yoga, which were significant in 7 trials.^[20,21,32,33,39,45,48]^

Three trials with 387 participants were included in the meditation trials subgroup (Fig. 3). These trials yielded non-significant results in favor of mediation. The overall analysis demonstrated a small significant effect (SMD = –0.22; 95% CI, –0.42 to –0.02; *P* = .03) and homogeneity (*I*^2^ = 0%, *P* = 1); meditation was slightly superior to control interventions for the reduction of activity limitation in the short term.

Two trials with 199 participants were included in the qigong trials subgroup (Fig. 3). One trial^[22]^ yielded significant results in favor of qigong; the other trial^[28]^ yielded significant results in favor of the control intervention. The overall analysis demonstrated no significant effect (*P* = .94) and a high degree of heterogeneity (*I*^2^ = 94%, *P* < .00001); qigong was as effective as control interventions for functional improvement.

Analysis of the pooled effect of walking and MBTs showed a significant small effect (SMD = –0.46; 95% CI, –0.73 to –0.18; *P* = .001) favoring these therapies over control interventions.

#### 3.3.3 Intermediate-term effects of interventions on pain

**Figure 4:**
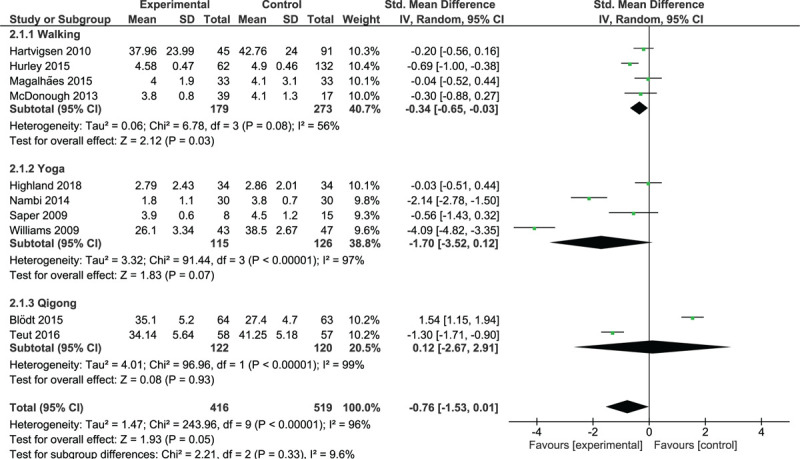
Intermediate-term effect of walking and MBT on pain.

Four trials involving 452 participants were included in the walking trials subgroup (Fig. 4). The analysis demonstrated a small, but significant, effect (SMD = –0.34; 95% CI, –0.65 to –0.03; *P* = .03) and a moderate degree of heterogeneity (*I*^2^ = 56%, *P* = .08); walking was slightly superior to control interventions in terms of pain improvement in the intermediate term. All trials yielded results in favor of walking, but only 1 trial^[30]^ showed that a daily walking program was statistically superior to weekly exercise or usual physiotherapy in terms of pain relief.

Four trials with 241 participants were included in the yoga trials subgroup (Fig. 4). The overall analysis demonstrated a non-significant effect (*P* = .07) and a high degree of heterogeneity (*I*^2^ = 97%, *P* < .00001); unlike in the short term, yoga was not superior to the control interventions in terms of pain relief in the intermediate term. All trials yielded results in favor of yoga, which were significant in 2 trials.^[34,45]^

Two trials with 242 participants were included in the qigong trials subgroup (Fig. 4). Teut et al^[18]^ reported significant results in favor of qigong, and Blödt et al^[28]^ reported significant results in favor of the control intervention. The overall analysis demonstrated a non-significant effect (*P* = .93) and a high degree of heterogeneity (*I*^2^ = 99%, *P* < .00001).

Analysis of the pooled effect of walking and MBTs showed a non-significant effect (SMD = –0.76; 95% CI, –1.53 to 0.01; *P* = .05) in favor of these therapies.

#### 3.3.4 Intermediate-term effects of interventions on activity limitation

**Figure 5:**
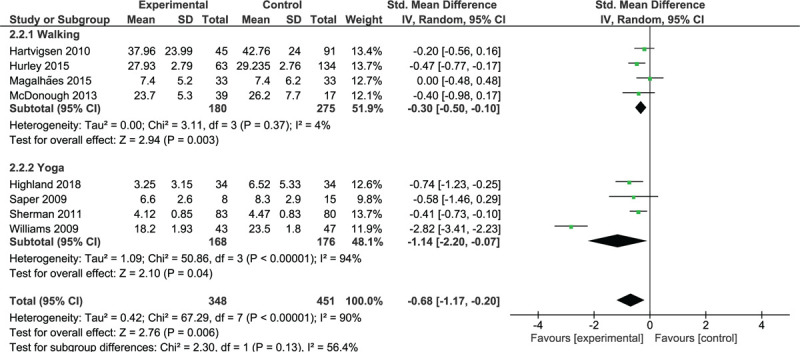
Intermediate-term effect of walking and MBT on activity limitations.

Four trials with 455 participants were included in the walking trials subgroup (Fig. 5). The overall analysis demonstrated a small significant effect (SMD = –0.30; 95% CI, –0.50 to –0.10; *P* = .003) in favor of walking and a lack of heterogeneity among trials (*I*^2^ = 4%, *P* = .37). One trial^[30]^ yielded a significant effect in favor of walking, and 2 trials^[36,43]^ yielded non-significant effects. One trial^[31]^ showed no effect of the addition of treadmill walking to participants exercise programs.

Four trials with 344 participants were included in the yoga trials subgroup (Fig. 5). All trials yielded results that favored yoga over the control interventions; these results were significant in 3 trials.^[20,42,45]^ The overall analysis demonstrated a significant effect (SMD = –1.14; 95% CI, –2.20 to –0.07; *P* = .04) and a high degree of heterogeneity (*I*^2^ = 94%, *P* < .00001).

Analysis of the pooled effect of walking and MBTs showed a significant moderate effect (SMD = –0.68; 95% CI, –1.17 to –0.20, *P* = .006) that favored these therapies over the control interventions.

### 3.4 Sensitivity analysis

**Figure 6:**
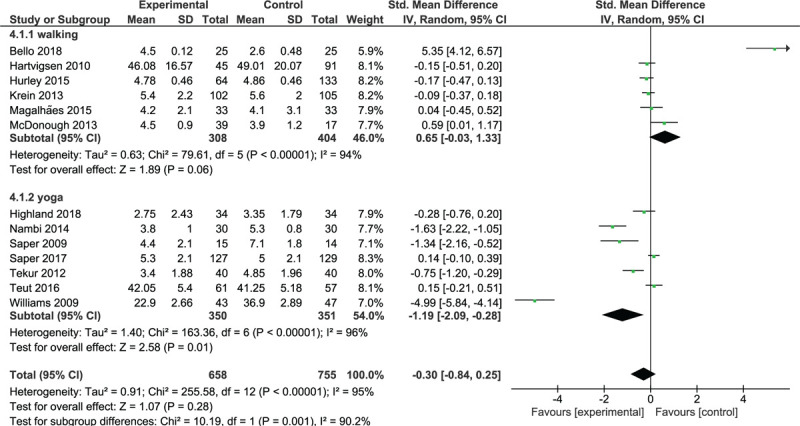
Short-term effect of walking and mind-body therapies on pain (sensitivity analysis).

Treatment significance effects of walking and MBTs remained the same across different analyses involving only trials with PEDro scores ≥5 (Fig. 6 and supplement 1–3).

## 4 Discussion

This systematic review and meta-analysis aimed to compare the effectiveness of walking and MBTs in CLBP management. Thirty one RCTs were included. The durations of walking and MBT interventions varied considerably, ranging from 7 days to 12 months; 77.4% of included trials used 6 to 12-week interventions. Overall, the findings showed that walking was as effective as control interventions in the short term and slightly superior in the intermediate term in reducing pain and activity limitation. MBTs, especially yoga, were more effective than control interventions in terms of pain and activity limitation, especially in the short term; the magnitude of the effect of yoga decreased over time.

We used subgroup analyses to compare the effectiveness of walking and MBTs. We compared only yoga to walking, given the similarity in the numbers of trials and in the control interventions used to examine these 2 therapies. In the short term, walking was as effective as control interventions in reducing pain and activity limitation, whereas yoga was more effective than control interventions. Thus, yoga can be considered to be more effective than walking for the reduction of pain and activity limitation in the short term. This finding is supported by previous demonstrations that yoga improves muscular strength, body flexibility, sleep patterns, overall well-being, and psychological symptoms among patients with LBP.^[49,50]^ Studies of non-LBP conditions have yielded results that are in line with our findings; Yekefallah et al^[51]^ found that yoga was more effective than walking for the reduction of blood glucose levels in patients with type 2 diabetes. Previous systematic reviews and meta-analyses^[52,53]^ have shown that yoga is more effective in the short term than in the long term, supporting our findings. The intermediate-term superiority of walking over yoga, especially for pain, in this study could reflect the insufficient home practice of yoga, whereas walking can be performed during daily activities. Walking is low cost, easily performed, and requires no special training or supervision.^[13]^ It helps patients with CLBP to maintain functional capabilities, and increases spine motor control and fitness levels.^[54]^ Walking also stimulates the brains release of serotonin and endorphins, which reduce pain and improve mood.^[55]^ Previous systematic reviews and meta-analyses^[10,56]^ support our finding of the effectiveness of walking for CLBP. Given our findings on their relative effectiveness in the short and intermediate terms, the combination of walking and yoga could be valuable therapeutic approach for CLBP. Dale and Stacey^[57]^ supported the multimodal treatment of chronic pain. They found moderate-quality evidence that multidisciplinary biopsychosocial rehabilitation treatment modestly improved pain compared with usual care or physical therapy alone.

Our results do not provide sufficient evidence to support the clinical effectiveness of meditation interventions in CLBP treatment, given the limited number of available trials. We found only that meditation was slightly superior to control interventions in reducing activity limitation in the short term. Current evidence shows no effect or a small effect of meditation in reducing pain and activity limitation in individuals with chronic pain, but high–quality evidence suggests that meditation reduces psychological symptoms.^[58,59]^ The effectiveness of meditation for CLBP can be explained by pain acceptance, which helps patients to improve their quality of life by decreasing pain intensity.^[60]^

Our ability to compare the effectiveness of tai chi and walking for CLBP was limited because only 2 trials examined the former. Within this limitation, tai chi appears to be superior to walking for the reduction of pain and activity limitation related to CLBP. The only trial included in our study that compared tai chi with walking^[39]^ showed that tai chi was more effective than backward walking in relieving pain in patients with non-specific CLBP. Studies of the effects of tai chi on other conditions have yielded results that are in line with our findings. Tai chi has been shown to have a more beneficial effect than brisk walking on cognitive function in older adults, and on strength, balance, and flexibility in sedentary women, due to its high cognitive demand.^[61,62]^ The practice of tai chi has also been shown to generate a more intense antioxidant effect than walking.^[63]^ The modes of action of tai chi are not completely understood; known effects include increased flexibility, structural mobility, endurance muscle strength, and cardiovascular function, as well as the reduction of psychological symptoms.^[64]^

Our findings demonstrated that qigong was not superior to control interventions in reducing pain and activity limitation in patients with CLBP. However, this conclusion should be taken cautiously, as we found only 3 studies that examined qigongs effectiveness. Some studies have compared walking and qigong in non-CLBP groups. A study conducted with adult women^[65]^ revealed the same effectiveness of walking and qigong, with a possible added effect of “qi” (the energy that powers body and spirit) that cannot be obtained with simple walking. A recent systematic review and meta-analysis^[66]^ suggested that Baduanjin, a type of qigong exercise, effectively reduces musculoskeletal pain and improves sleep quality among individuals with chronic illnesses.

### 4.1 Study limitations

Our findings need to be interpreted in the context of some specific limitations. First, only 1 of the 31 included trials compared walking with MBTs, and control interventions differed among trials. Second, we did not analyze fear avoidance or quality of life outcomes, as published in our protocol, as such assessments differed between walking and MBT trials. Some trials assessed overall quality of life, whereas others assessed its physical and mental components separately. The MBT trials did not evaluate fear avoidance. Third, 7 studies with missing data were excluded because we were unable to obtain these data by contacting the authors. The inclusion of data from these studies might have influenced the effects observed in our analysis. Finally, the search strategy was limited to full-length publications in English and French; thus, relevant publications in other languages (e.g., Chinese, Japanese, and Korean) were not included in this analysis.

## 5 Conclusion

This systematic review and meta-analysis aimed to compare the effectiveness of walking and MBTs in CLBP management. Our results suggest that MBTs, especially yoga, seem to be more effective in the short term, whereas walking seem to be more effective in the intermediate term. The combined use of MBTs and walking could fit the biopsychosocial model and could be a valuable treatment for CLBP in the short and long terms. Nevertheless, we suggest that further studies examine this combination and involve additional comparison of these interventions for the management of CLBP.

## Acknowledgments

Authors are grateful to « Association pour la Promotion de l’Éducation et de la Formation à l’Étranger (APEFE) » and « Wallonie-Bruxelles International (WBI) » for their financial support.

## Author contributions

**Conceptualization:** Ildephonse Nduwimana, Félix Nindorera, Jean-Louis Thonnard, and Oyéné Kossi.

**Data extraction and synthesis:** Ildephonse Nduwimana, Félix Nindorera.

**Development of the search strategy:** Ildephonse Nduwimana, Félix Nindorera, Oyéné Kossi.

**Protocol draft:** Ildephonse Nduwimana, Félix Nindorera, Jean-Louis Thonnard, Oyéné Kossi.

**Supervision:** Oyéné Kossi.

**Writing – original draft:** Ildephonse Nduwimana.

**Writing – review & editing:** Ildephonse Nduwimana, Félix Nindorera, Jean-Louis Thonnard, Oyéné Kossi.

## Supplementary Material

Supplemental Digital Content

## Supplementary Material

Supplemental Digital Content
