# Supplementary material for: Effectiveness of walking versus mind-body therapies in chronic low back pain: A systematic review and meta-analysis of recent randomized controlled trials
Source: Medicine (Baltimore). 2020 Aug 28;99(35):e21969. doi: 10.1097/MD.0000000000021969 (PMC7458239; doi:10.1097/MD.0000000000021969)

**Supplement 1:** Short-term effect of walking and MBT on activity limitations (sensitivity analysis)

**
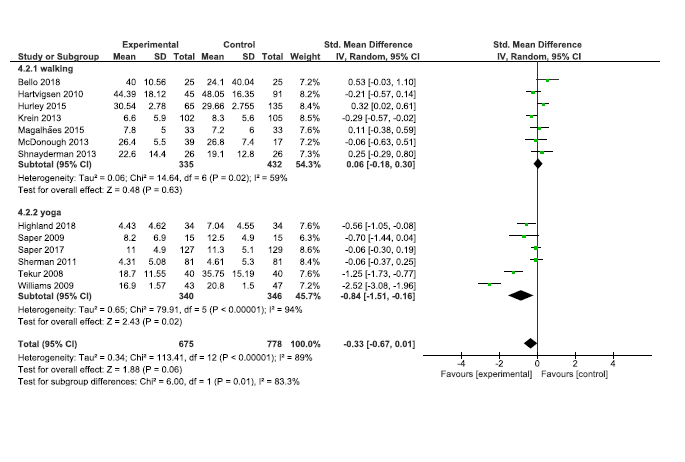
**

**Supplement 2:** Intermediate term effect of walking and MBT on pain (sensitivity analysis)


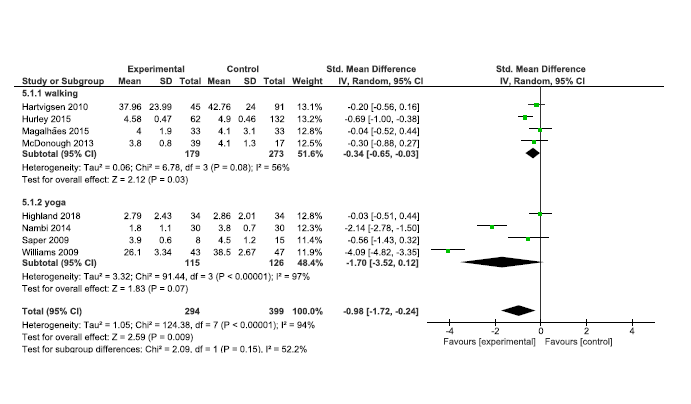


**Supplement 3**: Intermediate term effect of walking and MBT on activity limitations (sensitivity analysis)


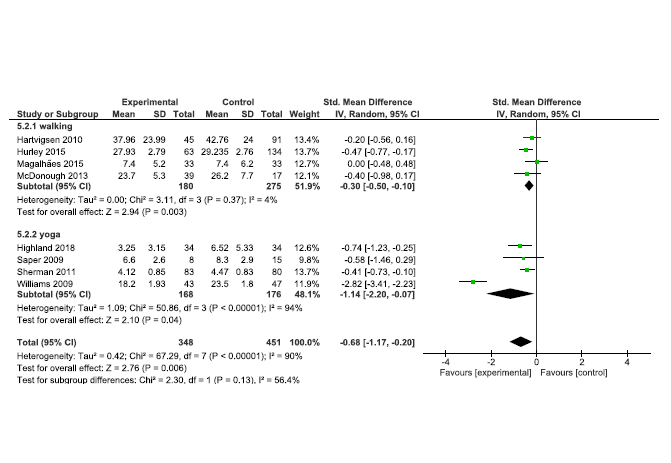

Supplement: Supplemental Digital Content [file medi-99-e21969-s001.doc]
