# Supplementary material for: Effectiveness of walking versus mind-body therapies in chronic low back pain: A systematic review and meta-analysis of recent randomized controlled trials
Source: Medicine (Baltimore). 2020 Aug 28;99(35):e21969. doi: 10.1097/MD.0000000000021969 (PMC7458239; doi:10.1097/MD.0000000000021969)
Supplement: Supplemental Digital Content [file medi-99-e21969-s002.docx]

**APPENDIX 1: Search strategies in databases**

**PubMed**

| #1  #2  #3  #4  #5  #6  #7  #8  #9  #10  #11 | "Low back pain"[MeSH Terms]  "recurrent low back pain"[Title/Abstract]) OR "low backache"[Title/Abstract]) OR "back pain" [Title/Abstract]) OR "lower back pain"[Title/Abstract]  #1 OR #2  walking[MeSH Terms]  walk*[Title/Abstract]) OR pedometer[Title/Abstract]) OR treadmill[Title/Abstract]  #4 OR #5  "mind body therapy"[MeSH Terms]  "mind body therapies"[Title/Abstract]) OR yoga[Title/Abstract]) OR "tai chi"[Title/Abstract]) OR "tai ji"[Title/Abstract]) OR "tai chi chuan"[Title/Abstract]) OR Qigong[Title/Abstract]) OR "relaxation therapy"[Title/Abstract]) OR relaxation[Title/Abstract]) OR meditation[Title/Abstract]) OR "mindfulness meditation" [Title/Abstract]) OR "breathing exercise"[Title/Abstract]) OR "mental healing"[Title/Abstract]  #7 OR #8  #6 OR #9  #3 AND #10 |
| --- | --- |

MeSH: Medical Subject Heading.

**Scopus**

{Low back pain} OR {recurrent low back pain} OR {low backache} OR {lower back pain} OR {lower back pain}

AND

Walking OR walk* OR pedometer OR treadmill OR {mind body therapy} OR {mind body therapies} OR yoga OR {tai chi} OR {tai ji} OR {tai chi chuan} OR Qigong OR {relaxation therapy} OR relaxation OR meditation OR {mindfulness meditation} OR {breathing exercise} OR {mental healing}

**Cochrane library**

| ID | Search Hits |
| --- | --- |
| #1 | ("Low back pain" OR "recurrent low back pain" OR "low backache" OR "back pain" OR "lower back pain"):ti,ab,kw (Word variations have been searched) |
| #2 | MeSH descriptor: [low back pain] explode all trees |
| #3 | walking OR walk* OR pedometer OR treadmill |
| #4 | MeSH descriptor: [Walking] explode all trees |
| #5 | "mind body therapies" OR yoga OR "tai chi" OR "tai ji" OR "tai chi chuan" OR Qigong OR "relaxation therapy" OR relaxation OR meditation OR "mindfulness meditation" OR "breathing exercise" OR "mental healing" |
| #6 | MeSH descriptor: [Mind-body therapy] explode all trees |
| #7 | #1 OR #2 |
| #8 | #3 OR #4 |
| #9 | #5 OR #6 |
| #10 | #8 OR #9 |
| #11 | #7 AND #10 |

**Sciences direct**

(“Low back pain”) AND (walking OR “mind body therapy” OR yoga OR “tai chi” OR Qigong OR “relaxation therapy” OR “meditation” OR “breathing exercise”)

**PsycInfo**

(Low back pain or recurrent low back pain or low back ache or lower back pain) AND (walking or gait or mind-body therapies or mind-body therapy or yoga or tai chi or relaxation therapy or breathing exercises or meditation or metal healing)
